# Making order from disorder in the nucleolus

**DOI:** 10.1101/gad.353467.125

**Published:** 2026-03-01

**Authors:** Susan J. Baserga

**Affiliations:** Department of Molecular Biophysics and Biochemistry, Department of Genetics, Department of Therapeutic Radiology, Yale School of Medicine, Yale University, New Haven, Connecticut 06520, USA

**Keywords:** intrinsically disordered regions, Nopp140, nucleolus, ribosomal RNA modification

## Abstract

The nucleolus and Cajal bodies in the eukaryotic nucleus serve as dynamic hubs for an intricate network of protein–protein and protein–RNA interactions that drive the macromolecular assembly of large and small ribonucleoproteins. However, the mechanisms by which these membrane-less organelles organize their components to fulfill these functions remain largely unknown. In this issue of *Genes & Development*, Meznad and colleagues (doi:10.1101/gad.353180.125) reveal that the nucleolar and Cajal body protein Nopp140 engages its protein partners through interactions mediated by intrinsically disordered regions. Together, they serve to build the web of interactions required for the tripartite organization of the nucleolus.

## Abstract (precis)

In this Outlook, Baserga discusses a study in this issue of *Genes & Development* by Meznad et al. that describes how ribonucleoprotein interactions mediated by protein chaperone Nopp140 drive nucleolar condensate formation and organization. Baserga highlights the central role of intrinsically disordered regions (IDRs) in potentiating the web of protein interactions critical for membrane-less organellar assembly and tripartite nucleolar organization.

Nucleoli and Cajal bodies were described as subnucleoplasmic organelles in eukaryotic cells more than 100 years ago, long before we knew their function in cellular homeostasis and growth. While both are lacking membranes, they are easily visible in the light microscope (Pederson 2011). We now know that the nucleolus (5 μm) is the site of transcription, processing, and assembly of ribosomes for their ultimate function in protein synthesis in the cytoplasm. Cajal bodies (0.1–1 μm) are the sites of small nuclear ribonucleoprotein (snRNP) and small nucleolar ribonucleoprotein (snoRNP) assembly. The outstanding question of how RNA–protein complexes, both large (ribosome) and small (snRNPs and snoRNPs), are assembled thus applies to both nuclear bodies. It is challenging to envision how potentially hundreds of protein–protein and protein–RNA interactions can be coordinated to produce these mature large and small RNPs.

One solution that seems likely is that the intrinsically disordered regions (IDRs) of the nuclear body proteins coordinate the multiple molecular interactions required for RNP assembly. IDRs, a subset of low-complexity regions of proteins, are defined as regions of proteins that do not have a structure, either as predicted through computational methods or experimentally determined (Holehouse and Kragelund 2024). Because they lack a stable structure, they can adopt many different shapes and interact with a wide variety of proteins to achieve structural plasticity. Nucleolar proteins are overrepresented in IDRs, with 20% of all nucleolar proteins (of 848) having a higher fraction of disordered residues per protein compared with 14% in cytosolic proteins (of 2054) (Stenström et al. 2020). Supporting the increased recognition of the key role that IDRs play in macromolecular assembly and the timeliness of this current study, the 2025 Albert Lasker Basic Medical Research Award was given to two pioneers in the field: Steven McKnight (University of Texas Southwestern) and Dirk Görlich (Max Planck Institute for Multidisciplinary Sciences, Göttingen) (Mittag 2025).

Nopp140 (HGNC name NOLC1 [nucleolar and coiled body phosphoprotein 1]) is a candidate for an IDR-containing protein that coordinates RNP assembly in the nucleolus and in Cajal bodies. Human Nopp140 exists as several protein isoforms of ∼699 amino acids. Remarkably, 80% of the protein is disordered (Uversky 2017). A disordered central repeat domain consisting of alternating acidic serine clusters and basic amino acids is highly phosphorylated by casein kinase 2. Nopp140 is localized to both nuclear bodies, linking the two (Isaac et al. 1998). It is known to interact with RNA polymerase I (RNAPI), which transcribes the pre-rRNA in the nucleolus. In the Cajal body, it interacts with the protein coilin (Courchaine et al. 2022) and small Cajal body-specific RNPs (scaRNPs), as well as snRNPs and snoRNPs.

In this issue of *Genes & Development*, Meznad et al. (2025) report multiple interactions of human Nopp140—via its intrinsically disordered regions (IDRs)—with other nucleolar proteins essential for ribosome biogenesis that also have IDRs, of which there are many. They demonstrate definitive protein–protein interactions via in vitro pull-down of recombinantly expressed purified proteins with a final analysis by SDS–polyacrylamide gel electrophoresis. They chose an exhaustive list of potential Nopp140-interacting nuclear body proteins with IDRs, including snoRNP proteins (NAP57 [DKC1; dyskerin], NOP56, NOP58, GAR1, and fibrillarin), a protein subunit of RNAPI (PAF49), and a nucleolar inhibitor of telomerase function (PINX1). They show that it is the Nopp140 IDR sequence repeats that bind to the IDRs in these interacting proteins. Nopp140 can also bridge interactions between RNAPI (PAF49) and the methylation guide snoRNP component NOP56, physically linking the steps of pre-rRNA transcription with the processing and RNA modification steps of ribosome biogenesis. Furthermore, the glycine–arginine-rich domains of GAR1 and fibrillarin serve as a new type of IDR. These in vitro results point to a role for Nopp140 in organizing nucleolar proteins via protein–protein interactions between their IDRs.

Additional in vivo results support the central role of Nopp140 in organizing nucleolar architecture. A tethering experiment in U2OS cells revealed that NOP58 and PAF49 can recruit Nopp140 in vivo as well. In addition, HAP1 cells expressing only a PAF49 missing its IDR disrupt the tripartite organization of the nucleolus. The nucleolus can be detected as three functional compartments in the electron microscope (EM): the fibrillar center (FC), the dense fibrillar component (DFC), and the granular component (GC). RNAPI transcription occurs in the FC and DFC interface, while pre-rRNA processing and modification take place in the DFC. The GC is the site of further preribosome assembly. Expressing PAF49 minus its IDR resulted in nucleoli lacking the three compartments by EM. This led to the major conclusion that Nopp140, interacting through its IDR with other IDR-containing snoRNP and RNAPI proteins, forms the basis of the liquid–liquid phase separation of the DFC of the nucleolus.

Unexpectedly, however, the PAF49 IDR is not required for its nucleolar function in ribosome biogenesis. HAP1 cells expressing only a PAF49 minus its IDR grow normally, consistent with the interpretation that the PAF49 IDR is not required for its function in pre-rRNA transcription. This suggests that although PAF49 uses its IDR to interact with Nopp140, disruption of this interaction is not sufficient to decrease RNAPI transcription. In addition, while Nopp140 bridges the interaction between PAF49 and methylation guide snoRNP component NOP56, expressing only PAF49 minus its IDR has no effect on 2′-O-methylation of the pre-rRNA. Taken together, these results lead to the conclusion that although the architecture of the nucleolus is disrupted when only PAF49 minus its IDR is expressed, that in itself does not affect the nucleolar function of making ribosomes.

Overall, this work is a biochemical tour de force that reveals Nopp140 as a central hub for protein–protein interactions in the nucleolus. The conclusions fit nicely with the emerging concept that it is the IDRs that mediate membrane-less organellar assembly. Furthermore, it provides an important piece of the puzzle in understanding how the tripartite organization of the nucleolus becomes apparent by electron microscopy.
